# Selenium-Nanoparticles-Loaded Chitosan/Chitooligosaccharide Microparticles and Their Antioxidant Potential: A Chemical and In Vivo Investigation

**DOI:** 10.3390/pharmaceutics12010043

**Published:** 2020-01-03

**Authors:** Kaikai Bai, Bihong Hong, Wenwen Huang, Jianlin He

**Affiliations:** 1 Third Institute of Oceanography, Ministry of Natural Resources, Xiamen 361005, China; bhhong@tio.org.cn (B.H.); wwhuang@tio.org.cn (W.H.); hejianlin@tio.org.cn (J.H.); 2 Technical Innovation Center for Utilization of Marine Biological Resources, Ministry of Natural Resources, Xiamen 361005, China

**Keywords:** selenium, nano, chitosan, chitooligosaccharide, alcohol, antioxidant

## Abstract

Selenium nanoparticles (SeNPs) have attracted attention due to their favorable properties, unique bioactivities, and potential for use in nutritional supplements and nanomedicine applications. However, the application of SeNPs in the clinic has been greatly hindered by their poor stability, and their potential to protect against alcohol-induced oxidative stress has not been fully investigated. Herein, SeNPs were synthesized in the presence of chitosan (CS) or chitooligosaccharide (COS), and a mixture of SeNPs, CS, and COS was spray-dried to prepare selenium-nanoparticles-loaded chitosan/chitooligosaccharide microparticles (SeNPs-CS/COS-Ms). Their physicochemical properties, including morphology, elemental state, size distribution, surface potential, and characteristic structure, were investigated. The release of SeNPs from the vehicle and the free radical scavenging ability of SeNPs-CS/COS-Ms were also studied. Furthermore, the safety of SeNPs-CS/COS-Ms and their antioxidant activity against alcohol were evaluated in mice. The results indicate that SeNPs-CS/COS-Ms, with a novel structure characterized by their smooth or wrinkled surface, hollow core, and COS body filled with SeNPs-CS nanobeads, were able to release SeNPs and scavenge DPPH and superoxide anion radicals. SeNPs-CS/COS-Ms were found to be much safer than selenite, and they might protect mice from ethanol-induced oxidative stress by reducing lipid and protein oxidation and by boosting glutathione peroxidase (GSH-Px), superoxide dismutase (SOD), and catalase (CAT). In conclusion, SeNPs-CS/COS-Ms offer a new way to develop stable SeNPs with higher efficacy and better biosafety, and the antioxidant potential of SeNPs-CS/COS-Ms against ethanol deserves further development.

## 1. Introduction

As a dietary nutrient, selenium (Se) is an indispensable trace element required for animals and humans, and plays an essential role in many aspects of health [1,2]. To meet the daily requirement of Se, Se supplementation is necessary, especially in those suffering from Se deficiency [1,2,3]. In recent years, selenium nanoparticles (SeNPs), a unique type of elemental selenium of nano-defined size, have attracted attention due to their favorable properties and excellent biological activities, such as scavenging free radicals [4,5], antibacterial activity [4,6], anti-tumor activity [4,7], promoting animal growth [4,8], boosting Se retention [4,8,9], and enhancing oxidant status in vivo [4,9,10,11]. These nanoparticles seem to be more efficient than many Se compounds in boosting selenoenzymes, as they have lower acute toxicity as well as acceptable bioavailability [2,9,10,11,12,13]. SeNPs are regarded as a prospective Se supplement due to their potential for use in nutritional supplements, chemoprevention, chemical therapy against cancer, and other nanomedicine applications [4,10,11].

The antioxidant activity of SeNPs plays an important part in the bioactivities of these nanoparticles, since Se is an integral part of the catalytic site of at least 25 human selenoproteins and enzymes and plays a role in protecting cells from oxidative injury [2,4]. Se deficiency might break the balance between oxidants and antioxidants in the body, which may increase oxidation-associated risks, especially when the body is challenged by oxidative stress [1,2,4,10,11,12,13,14,15]. However, animals and humans continue to suffer from various forms of oxidative damage induced by chemicals [16,17] or emotional stress [18,19]. Among the oxidation-associated risks, excessive consumption of alcohol can induce oxidative stress in the body [17,20], and Se deficiency can increase alcohol-induced oxidative stress in tissues [10,11,17,21]. However, the protective potential of SeNPs against alcohol-induced oxidative challenge has yet to be reported. In addition, the commercial application of SeNPs in oral administration systems has been hindered by their poor stability [10,11]. A feasible formulation of SeNPs needs to be developed.

Chitosan (CS) is the only positively charged natural polysaccharide and has excellent biodegradability and biocompatibility [22]. The polysaccharide and its derivatives have been extensively examined in the pharmaceutical industry due to their potential in the development of medicine delivery systems [22]. In this study, SeNPs were quickly synthesized in CS, and a mixture of SeNPs, CS, and chitooligosaccharide (COS) was spray-dried to produce selenium-nanoparticles-loaded chitosan/chitooligosaccharide microparticles (SeNPs-CS/COS-Ms). The structure, SeNP release performance, free radical scavenging ability, and biosafety of the SeNPs-CS/COS-Ms were investigated, and the antioxidant potential of this new Se formulation was also studied in mice given alcohol. The authors hope that the results of this study will assist with the development of a SeNP-based Se supplement.

## 2. Materials and Methods

### 2.1. Materials and Animals

#### 2.1.1. Materials

Food-grade CS (90.32% deacetylated, an average molecular weight of 37 kDa) and COS (an average molecular weight of 2.5 kDa) were purchased from Aoxin Pharmaceutical Co. Ltd. (Taizhou, China). DPPH (1,1-diphenyl-2-picrylhydrazyl), pepsin from porcine gastric mucosa (≥250 units/mg solid), pancreatin from porcine pancreas (8 × United States Pharmacopoeia (USP) specifications), and pyrogallic acid of high purity were obtained from Sigma-Aldrich (St.Lousi, MO, USA). Reagents of food grade, including acetic acid, sodium selenite, and ascorbic acid (Vc), and other reagents of analytical grade, were purchased from commercial suppliers (Sinopharm Chemical Reagent Co., Ltd., Shanghai, China). The assay kits for measuring thiobarbituric-acid-reactive substances (TBARS), glutathione (GSH), glutathione peroxidase (GSH-Px), superoxide dismutase (SOD), catalase (CAT), total carbonyl compounds (TCCs), and protein content were provided by Jiancheng Bio-engineering Institute (Nanjing, China).

#### 2.1.2. Animals

Kunming (KM) mice of specific-pathogen-free (SPF) grade, half male and half female, 8–10 weeks old, and weighing 18–22 g, were supplied and housed by the Laboratory Animal Center, Shenyang Pharmaceutical University (Shenyang, China) with the license No. SCXK (Liaoning) 2015-0001. In addition, male KM mice of SPF grade, 8–10 weeks old, and 20–25 g in body weight (bw) were purchased from Beijing HFK Bioscience Co., Ltd. with the license No. SCXK (Beijing) 2014-0004, and were housed in a standardized sterile animal room located at the Fujian Health College (Fuzhou, China) with a controlled temperature (25 ± 2 °C) and humidity (50 ± 10%) and a 12-h light/dark cycle. The procedures that were utilized in the animal experiments were approved by the Animal Ethics Committees at Shenyang Pharmaceutical University (ethical committee approval number: SYPU-IACUC-C2017-4-21-208, Date (21/4/2017)) and Fujian Health College (ethical committee approval number: DW2018112501, Date (25/11/2018)). They were also compliant with the provisions and general recommendations of the Chinese Experimental Animals Administration Legislation.

### 2.2. Chemicals and Characterization

#### 2.2.1. Synthesis and Characterization of SeNPs

SeNPs were synthesized in the presence of CS or COS as described in previous studies [10,11] with little modification. Chitosan-decorated selenium nanoparticles (CS-SeNPs) were dialyzed against 1% (*w*/*w*) acetic acid to remove the excess Vc and other by-products. The purification of CS-SeNPs was conducted by means of ultrafiltration (UF) in an acetic acid solution, unless the permeate did not fade in 1 μmol/L KMnO_4_ solution within 20 min. The morphological characteristics of SeNPs were observed by using a TEM (JEM-2100; JEOL, Tokyo, Japan) device equipped with an energy-dispersive X-ray spectroscopy (EDS) machine as previously stated [10,11]. The size distribution and the zeta-potential of SeNPs were measured by using a Zetasizer Nano ZS particle analyzer (VEM3600; Malvern Instruments, Malvern, UK) with a 173° scattering angle as previously described [10,11].

#### 2.2.2. Preparation and Characterization of SeNPs-CS/COS-Ms

The purified CS-SeNPs were mixed with COS or additional CS, and the mixture was spray-dried using a laboratory spray dryer (SY-6000; Shiyuan Biological Equipment Engineering Co. Ltd., Shanghai, China) with a standard 0.7 mm nozzle to generate SeNPs-CS/COS-Ms. The proportions of Se, CS, and COS could be adjusted by modifying the material ratio, which also allowed for the preparation of CS/COS microparticles (namely CS/COS-Ms) without SeNPs. The Se content of SeNPs-CS/COS-Ms was determined by utilizing inductively coupled plasma mass spectrometry (ICP-MS) [23].

The morphology of SeNPs-CS/COS-Ms was observed by an SEM (S-4800; Hitachi, Tokyo, Japan) device. The size distribution of these microspheres was measured by using a particulate size analyzer (LS-POP(6); Zhuhai OMIC Instruments Co. Ltd., Zhuhai, China) [10,11]. In order to explore structural details, the ultrasonic disruption of SeNPs-CS/COS-Ms was performed in absolute ethanol or 50% (*v*/*v*) ethanol before SEM observations were made. Moreover, a Nicolet Nexus 470 spectrometer (Thermo Fisher Scientific, Waltham, MA, USA) was used to determine the FTIR spectra, which were acquired at 400–4000 cm^−1^ with a 4 cm^−1^ resolution [10,11]. In addition, an XPS measurement was performed using a high-resolution photoelectron spectrograph (Escalab 250Xi; Thermo Fisher Scientific, Waltham, MA, USA) equipped with a monochromatic Al *K_α_* X-ray source and a dual-beam charge neutralization system composed of a low-energy electron flood gun (~1 eV) and an argon ion gun (≤10 eV) [10,11].

### 2.3. Release Experiment

The release of SeNPs from SeNPs-CS/COS-M-C was evaluated in simulated gastric fluid (SGF) [24] composed of HCl solution (pH 1.5) with 0.1% (*w*/*v*) pepsin and simulated intestinal fluid (SIF) [25] composed of phosphate-buffered saline (PBS) (pH 7.2) with 1.0% (*w*/*v*) pancreatin. Briefly, 0.1 g of SeNPs-CS/COS-Ms was placed in 100 mL of SGF or SIF, and the mixture was stirred at 100 rpm and incubated at 37 °C. At certain time intervals, 1 mL of release medium was collected and 1 mL fresh SGF or SIF was placed in the release system. The medium was centrifuged (1000× *g*, 1 min), and then the supernatant was filtrated by using a PES filter membrane (with a pore size of 0.22 µm) to obtain the filtrate containing released SeNPs. The Se content of the filtrate was measured by an ICP-MS assay according to the method of Dufailly [23]. The Se contents were plotted as a function of time. All experiments were performed in triplicate.

### 2.4. Free Radical Scavenging Tests

#### 2.4.1. DPPH Radical (•DPPH) Scavenging Assay

The •DPPH scavenging assay was performed according to the method of Zhai [26] and Xu [27], with a slight modification. In brief, a sample was mixed with 1 mL of DPPH ethanol solution (1 mM), and the mixture was adjusted to 10 mL using ethanol. After incubation for 30 min at 25 °C, the absorbance of the mixture was measured at 517 nm. The DPPH radical scavenging ability was calculated as follows:

Scavenging ability (•DPPH) = (A_c_ − A_a_ + A_b_)/A_c_ × 100% (1)

where A_c_ is the absorbance of DPPH without a sample; A_a_ is the absorbance of the sample mixed with DPPH solution; and A_b_ is the absorbance of the sample without DPPH solution.

#### 2.4.2. Superoxide Anion Radical (•O_2_^−^) Scavenging Assay

The influence of a sample on the generation of •O_2_^−^ by pyrogallic acid [28] was determined by using a spectrophotometric method with some modifications. Tris-HCl buffer (50 mM, pH 8.2) containing certain concentrations of the tested sample was incubated at 25 °C for 30 min. Subsequently, it was mixed with pyrogallic acid (0.75 mM) and rapidly shaken. The mixture was quickly adjusted to the same volume, and its absorbance was recorded in succession using a spectrophotometer (UV-1780, Shimadzu, Kyoto, Japan) at 320 nm. The slope value of each absorbance curve was measured through linear regression. The ability to scavenge •O_2_^−^ was calculated as follows:

Scavenging ability (•O_2_^−^) = (S_a_ − S_b_)/S_a_ × 100% (2)

where S_a_ was the slope rate when the sample was mixed with pyrogallic acid; and S_b_ was the slope rate when only pyrogallic acid was used.

### 2.5. Animal Experiment

#### 2.5.1. Acute Lethal Test In Vivo

The acute lethal property of SeNPs-CS/COS-Ms (CS:COS = 10:90 *w*/*w*, containing 15.1 mg Se/kg) was determined as previously described [10,11] with little modification. Briefly, 50 KM mice were randomly divided into five groups with 10 mice per group after adaptation for 3 days. Each group was given SeNPs-CS/COS-Ms (10.00, 7.50, 5.62, 4.20, and 3.16 g/kg bw, respectively) by a single intragastric administration, and cumulative mortality within 14 days was recorded to calculate the median lethal dose (or LD_50_) by the Bliss method [29]. Another group of 40 KM mice was given CS/COS-M (CS:COS = 10:90 *w*/*w*) at a cumulative dose of 20 g/kg bw within 12 h to evaluate the acute toxicity of this vehicle for SeNPs.

#### 2.5.2. Ethanol Challenge Test

An alcohol challenge test was performed as described by Han [30] and Zeng [31] with some modifications. In brief, 80 male KM mice were randomly divided into eight groups (10 mice per group) (Control, Model, Vc, Selenite, CS/COS-M (CS:COS = 10:90), and three SeNPs-CS/COS-Ms (CS:COS = 10:90, containing 15.1 mg Se/kg) groups (L-Se, M-Se, and H-Se)). Each group was administered a dose daily by a gavage for five consecutive weeks, as shown in Table 1, and body weight was recorded throughout the whole experiment. Except for the normal control, mice were deprived of food after the final pretreatment, but were allowed free access to water. Sixteen hours later, normal control mice were given an equal volume of water and the fasting mice were intragastrically administered a single dose of 50% ethanol (4.8 g/kg bw, namely 12 mL/kg bw). Six hours later, the mice were sacrificed, the blood was collected into heparin-free tubes to obtain serum, and the liver was also obtained. The Se content in serum was determined by an ICP-MS assay [23]. Then, the GSH-Px, SOD, CAT, TBARS, GSH, and TCC levels in serum or in liver were measured, following the instructions in the commercial kits.

### 2.6. Statistical Analysis

In all of the experiments, data are presented as mean ± SD. The difference between two groups was analyzed by a Student’s *t*-test involving the utilization of the SPSS software program (version 17.0 for Windows). A *p* value of less than 0.05 was considered to be statistically significant.

## 3. Results and Discussion

### 3.1. Characterization of SeNPs

**Figure 1:**
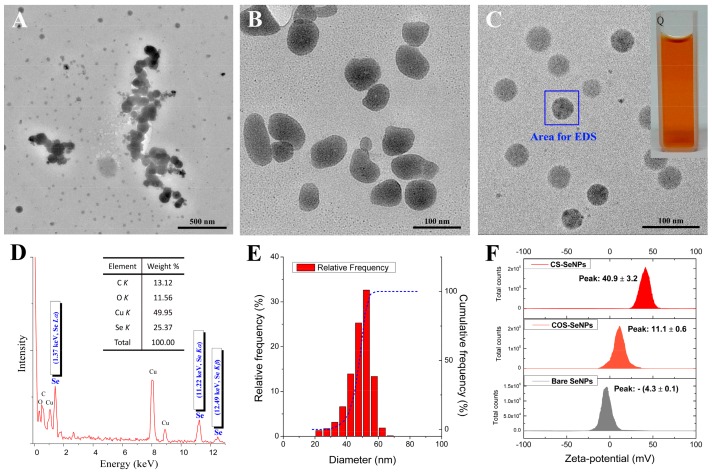
Typical morphology and formation of selenium nanoparticles (SeNPs). TEM images of (**A**) bare SeNPs, (**B**) chitooligosaccharide (COS)-SeNPs, and (**C**) chitosan (CS)-SeNPs. (**D**) EDS spectra of CS-SeNPs determined from the area shown in Figure 1C. (**E**) The size distribution of CS-SeNPs measured from the TEM results. (**F**) Zeta-potentials of bare SeNPs, COS-SeNPs, and CS-SeNPs, measured at a pH of 5.0 ± 0.3.

Polysaccharides such as CS [10,11,32], alginate [33], sialic acid [34], and gum arabic [35] have been utilized to synthesize and stabilize SeNPs, and SeNPs with different morphologies have been obtained. It seems that polysaccharides of high molecular weight (MW) can be used to prepare and stabilize SeNPs. Herein, aqueous Se (IV) was chemically reduced by ascorbic acid (Vc) to synthesize SeNPs (elemental Se particles) in the presence of CS (37 kDa) or COS (2.5 kDa) in order to evaluate the surface decoration of SeNPs with the positively charged polysaccharides. As a result, spherical SeNPs were initially synthesized in CS, COS, or water, though their size varied (Figure S1A–C, Supplementary Materials). However, the changes in them differed. A few hours later, the bare SeNPs that were synthesized in water without any surface decoration quickly aggregated into bulks (Figure 1A), while a change in shape and aggregation of SeNPs were found in the COS solution at 24 h after the initial formation stage (Figure 1B). However, highly uniform monodisperse spherical SeNPs, as presented in Figure 1C, were finally obtained in aqueous CS, with an orange or red appearance that was dependent on the concentration of SeNPs (Figure 1C inset). The chitosan-decorated selenium nanoparticles (CS-SeNPs) were found to remain stable at 4 °C for about 1 month without visible deposition. CS was found to be superior to COS in terms of controlling the formation, shape, and stability of SeNPs.

Some characteristics of SeNPs were also investigated. Typical Se peaks (1.37, 11.22, and 12.49 keV, identified as Se *L_α_*, Se *K_α_*, and Se *K_β_*, respectively) were found in the EDS spectra of SeNPs, confirming the elemental nature of these nanoparticles (Figure 1D). Moreover, a size distribution analysis indicated that the diameter of SeNPs could be controlled by CS within a nano-defined range (Figure 1E and Table 2). Nonetheless, the size distribution as measured by TEM (Figure 1E) and dynamic light scattering (DLS, Table 2) showed that the average diameters of the CS-SeNPs were around 50 and 100 nm, respectively. These results suggest that an invisible layer covered the SeNPs since DLS can only measure hydrodynamic size [10,32]. Furthermore, the size and dispersity of the SeNPs were monitored over time to evaluate the stability of these nanoparticles in solution. As shown in Table 2, the fresh SeNPs had similar diameter and polydispersity index (PDI) values. The Z-average sizes of the bare SeNPs and COS-SeNPs increased over time; however, the size of the CS-SeNPs was found to be about 80 nm with a low PDI (0.116 ± 0.018) after 14 days of storage. The ‘bottom-up’ growth process of COS-SeNPs and the ‘top-down’ shrinkage process of CS-SeNPs over time reported by Leng et al. [32] were also found in this study, indicating that CS was able to control the size of SeNPs during common storage. The ability of CS to control the stability of SeNPs is important to the industrial production of SeNPs.

In addition, the zeta-potential was studied at a pH of 5.0 ± 0.3. The results shown in Figure 1F illustrate that the surface potential of the SeNPs was dependent on the surface decoration. To be more specific, the bare SeNPs were almost electroneutral (−4.3 ± 0.1 mV) in water, while the zeta-potential of SeNPs was elevated by CS to 40.9 ± 3.2 mV, probably due to the positively charged –NH_3_^+^ groups from the CS [10,32]. This might ensure that SeNPs remain stable during the process of dialysis or UF. COS decoration also led to an increase in the zeta-potential of the SeNPs (11.1 ± 0.6 mV); however, the elevation was weak and partly responsible for the weak stability of SeNPs in COS solution. Nonetheless, these results may be not be in line with a previous study [32] that reported higher stability (30 days) and zeta-potentials (>45 mV) for SeNPs when these nanoparticles were modulated by CS (3 and 200 kDa) at a pH of 3.2 ± 0.3. This result could be partly attributed to the fact that the zeta-potential might change with pH [35]. A lower pH may result in a higher zeta-potential, as more –NH_2_ groups might become –NH_3_^+^ groups, which may be beneficial to the stability of SeNPs. A previous study [32] reported that CS was more efficient than COS at elevating the zeta-potential of SeNPs. Considering the relationship between nanoparticles’ stability and their zeta-potential, CS was found to be superior to COS in the preparation of stable SeNPs.

### 3.2. Characterization of SeNPs-CS/COS-Ms

Bare SeNPs and CS-SeNPs are not available for commercial application in oral administration systems due to their poor stability [10,11], which is closely related to the decrease in zeta-potential during storage. SeNPs might finally become dark bulk aggregates even in the presence of CS, following an unavoidable enlargement in size [10,36] and aggregation in an aqueous environment [10,11,36]. In our previous work, SeNPs were loaded into solid CS microspheres [10] or a chitosan/citrate complex [11] to overcome the instability of the nanoparticles, and these solid mixtures allowed for the release of SeNPs and Se supplementation in animals. Physical isolation of SeNPs in solid vehicles seems to be a feasible way to develop a Se supplement based on SeNPs [10,11]. However, more efficient SeNP supplementation is needed.

**Figure 2:**
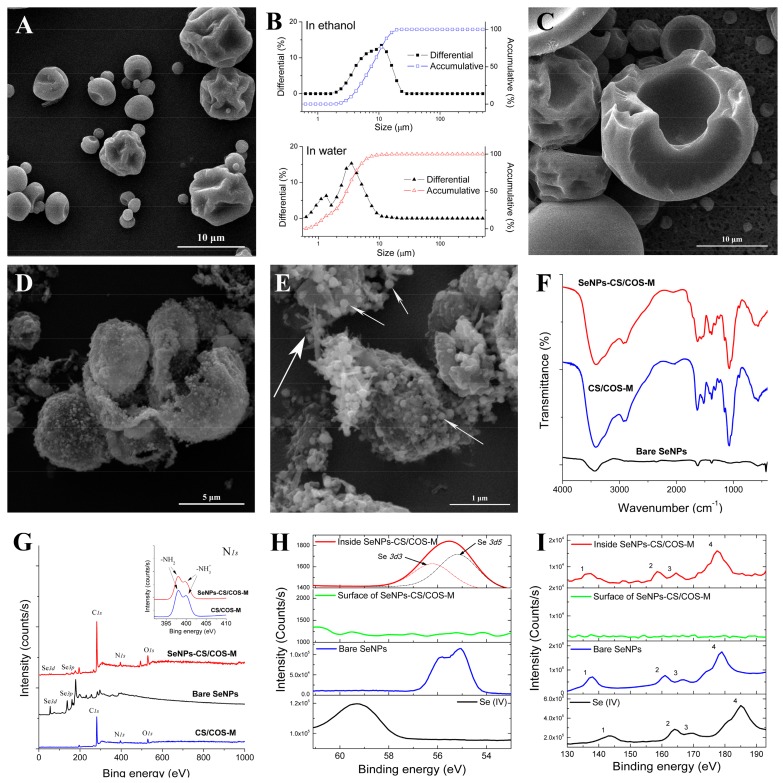
The typical morphology and chemical properties of selenium-nanoparticles-loaded chitosan/chitooligosaccharide microparticles (SeNPs-CS/COS-Ms). (**A**) An SEM image of SeNPs-CS/COS-Ms. (**B**) The size distribution of SeNPs-CS/COS-Ms in ethanol (upper) and in water (lower). (**C**) An SEM image of SeNPs-CS/COS-Ms after ultrasonic disruption in absolute ethanol. (**D**–**E**) SEM images of SeNPs-CS/COS-Ms at different magnifications, exactly (**D**) 16,000× and (**E**) 50,000×, after ultrasonic disruption in 50% ethanol. (**F**) FTIR spectra of bare SeNPs, CS/COS, and SeNPs-CS/COS-Ms. (G–I) XPS results, including (**G**) wide-range XPS patterns, (**H**) Se *3d* scan XPS patterns composed of Se *3d3* and Se *3d5* signals, and (**I**) Se *3p* scan XPS patterns. In Panel (**E**), smaller arrows indicate CS-SeNP nanobeads, while the bigger one represents the linear CS structure.

COS was found to be more soluble than CS because of its low MW, while CS was found to be more efficient than COS at maintaining stable SeNPs. In the present study, SeNPs, CS, and COS were mixed together in a proper proportion and the mixture was spray-dried to produce SeNPs-CS/COS-Ms. The SeNPs complex is actually a collection of microparticles with a spherical or irregularly wrinkled surface (Figure 2A). The size distribution analysis performed in ethanol indicated that the size of the microparticles was 2–20 μm, while small sizes were observed in water (Figure 2B). Neither of the polysaccharides were soluble in ethanol, while COS was found to be soluble in water and CS was found to be not soluble in water [22,37,38]. Moreover, ultrasonic disruption of particles was conducted in absolute ethanol, and it was found that the SeNPs-CS/COS-Ms were hollow (Figure 2C). The structure was very different from that of selenium-nanoparticles-loaded chitosan microspheres (SeNPs-CS-Ms) with solid cores [10]. The wrinkled surface may partly be explained by the lack of balance between the moisture diffusion and the moisture evaporation that occurred during spray-drying [39], and partly by the weak mechanical strength of COS (and also the low MW of CS) as compared with CS [40]. As for the hollow core, this may have resulted from the film-formation capacity and the viscosity of the polysaccharides [37,38], which allowed for the generation of a smooth surface inside SeNPs-CS/COS-Ms. Evidently, SeNPs-CS/COS-Ms with an appreciable specific surface area were developed.

Furthermore, 50% ethanol was used to remove the COS within SeNPs-CS/COS-Ms in order to obtain more detail. The skeleton, comprised of CS and SeNPs, remained as presented in Figure 2D, since these two compounds are unable to be dissolved by 50% ethanol in theory. Nanobeads with a size of 60–150 nm were found on the remnants of the SeNPs-CS/COS-Ms, and seemed to be fixed onto some linear or lumpy structures (Figure 2E). This suggests that the space around the nanobeads was filled with COS. That is, the hollow and wrinkled SeNPs-CS/COS-Ms were actually a close layer of solid COS filled with the nanobeads. This allowed for the quick disintegration of SeNPs-CS/COS-Ms in water as shown in Figure 2B, which is impossible for SeNPs-CS-Ms in the same dispersant [10]. The nanobeads, whose EDS patterns were similar to that shown in Figure 1D, showed that individual SeNPs were capped by CS rather than COS during the mixing and spray-drying processes. This also implies that CS is superior to COS in terms of the surface decoration of SeNPs. The structure of SeNPs-CS/COS-Ms might facilitate the physical isolation and release of SeNPs.

Other chemical characteristics of the SeNPs-CS/COS-Ms were investigated. A FTIR analysis was conducted to explore whether any chemical modification of functional groups occurred. SeNPs-CS/COS-Ms with different material ratios were found to share comparable FTIR spectra, and they seemed to be a simple collection of SeNPs, CS, and COS, without any new functional groups found in the FTIR spectra (Figure 2F). In addition, an XPS analysis was performed to examine the status of Se. As shown in Figure 2G, the XPS signals of C, O, N, and Se were found, and the characteristic peaks of –NH_3_^+^ and –NH_2_ were found in both CS/COS-M and SeNPs-CS/COS-M, which is consistent with previous studies reporting that these two chemical states of nitrogen can be observed by N *1s* XPS [10,11,41]. Additionally, the peaks found at 55.3 eV and 59.5 eV in the XPS patterns (shown in Figure 2H) were identified as the typical Se *3d* signals of Se (0) and Se (IV), respectively, confirming that the Se within the SeNPs-CS/COS-Ms was in an elementary state [10,11,32]. The Se *3d* result observed in the SeNPs-CS/COS-Ms was actually composed of the Se *3d3* and Se *3d5* signals, which was also found in the bare SeNPs (Figure 2H). This result implies that these chemically fabricated SeNPs are similar in terms of their molecular structure and chemical environment. However, the Se *3d* signals on the surface of the SeNPs-CS/COS-Ms were much weaker than those inside the microparticles, which were found by using argon ion etching to expose the elements inside. Similar results were observed when Se *3p* scanning was conducted, and a shift in the Se *3p* signals was also found when comparing Se (0) with Se (IV) (Figure 2I). Undoubtedly, most of the SeNPs were enclosed inside SeNPs-CS/COS-Ms.

### 3.3. Release of SeNPs from SeNPs-CS/COS-Ms

**Figure 3:**
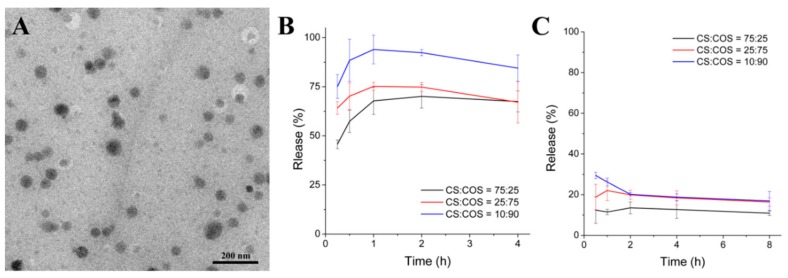
The release of SeNPs from SeNPs-CS/COS-Ms in simulated gastric fluid (SGF) and simulated intestinal fluid (SIF). (**A**) A typical TEM image of the released SeNPs in SGF or SIF. (**B**,**C**) The relative release rates of SeNPs from SeNPs-CS/COS-Ms with different material ratios of CS:COS (75:25, 25:75, and 10:90) in (**B**) SGF and (**C**) SIF.

The release of nanoparticles from a vehicle is very important to their absorption and their bioactivities in vivo [4,11]. In the previous studies [10,11] performed by the authors, SeNPs were found to escape from their vehicles, including CS microspheres and a chitosan/citrate complex, finally resulting in Se supplementation. Nonetheless, aspects of the vehicle’s composition, such as the CS to COS ratio, might affect the release of SeNPs from the vehicle in water or the digestive tract. In this study, the release of SeNPs from SeNPs-CS/COS-Ms was tested in SGF (pH 1.5) and SIF (pH 7.2) by using some SeNPs-CS/COS-M samples with comparable Se content (approximately 15 mg/g) and different COS:CS ratios (25:75, 75:25, and 90:10). After incubation in SGF or SIF at 37 °C, intact SeNPs could be found in the digestive fluids (Figure 3A), suggesting that SeNPs were released from SeNPs-CS/COS-Ms in the mammalian digestive tract. This result also implies that embedding SeNPs in CS/COS microspheres does not significantly modify SeNPs’ size and morphology, which are two important properties that might profoundly affect the bioactivities of the nanoparticles [5,42].

The release of SeNPs was found to be dependent on the digestive environment and the CS:COS ratio. The release of SeNPs was found to be faster in SGF than in SIF (Figure 3B,C). This result may be due to the acid solubility of CS, which allowed for the full release of SeNPs, while only the SeNPs absorbed on the surface or those embedded by COS could escape in the SIF. This indicates that most of the SeNPs will be released in the stomach but not in the gut. In addition, the release of SeNPs was affected by the CS:COS ratio in the order 10:90 > 25:75 > 75:25 (Figure 3B). This suggests that a higher percentage of COS in SeNPs-CS/COS-Ms might benefit the release of SeNPs in the stomach, as COS is freely soluble in water in a wide pH range [37,38]. The wrinkled, hollow, and nanobead-loaded structure of SeNPs-CS/COS-Ms might also have contributed to the different release rates of SeNPs, because the CS-SeNP nanobeads were surrounded by COS. An increase in the COS content and the quick dissolution of COS might be beneficial to the exposure of the SeNPs to the digestive system. Moreover, a decrease in Se release was found in both SGF and SIF, though the decrease was very slight. This result indicates that the stability of SeNPs might be challenged by digestive fluids. When considering the residence period (commonly 1–4 h, dependent on food) of the diet in the stomach [43,44], most of the released SeNPs, however, might be stable in the gastric phase. SeNPs-CS/COS-Ms with a CS:COS ratio of ≤10:90, which enables the full release of SeNPs in a gastric environment, were optimized for further investigations.

### 3.4. Free Radical Scavenging Ability of SeNPs-CS/COS-Ms

**Figure 4:**
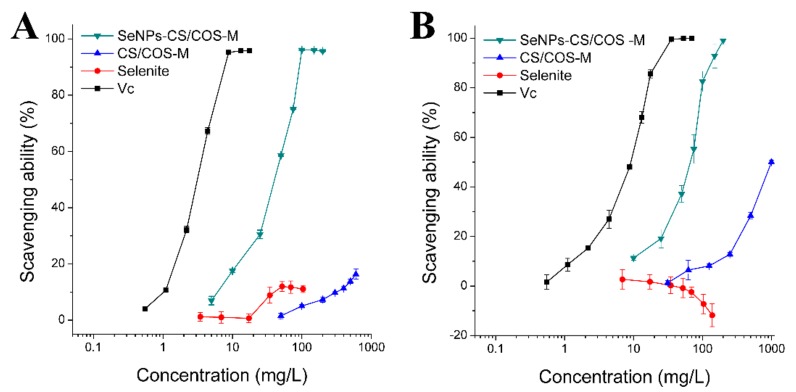
Free radical scavenging abilities against (**A**) DPPH (•DPPH) and (**B**) superoxide anion (•O_2_^−^) radicals. SeNPs-CS/COS-Ms containing 15 mg Se/kg (CS:COS = 10:90) and CS/COS-Ms (CS:COS = 10:90) were evaluated in this section.

Overconsumption of alcohol can produce an excessive amount of radical oxygen species (ROS), which can lead to oxidative damage to the lipids and proteins in tissues [17,20]. Here, •DPPH and •O_2_^−^ were used as model free radicals to investigate the radical scavenging ability of SeNPs-CS/COS-Ms. Considering the acceptable release of SeNPs-CS/COS-Ms when their COS:CS ratio is high, SeNPs-CS/COS-Ms with a CS:COS ratio of 10:90 were tested. As a strong antioxidant, Vc exhibited potent radical scavenging activity against these two free radicals, while selenite did not remove the radicals (Figure 4A,B). However, SeNPs-CS/COS-Ms were able to clear •DPPH and •O_2_^−^ in a dose-dependent manner, although their capacity to scavenging radicals was weaker than that of Vc (Table 3). The free radical scavenging capacity of SeNPs-CS/COS-Ms, which was found to be equal to approximately 1/10 (•DPPH) or 1/6 (•O_2_^−^) of that of Vc, was acceptable.

The total free radical scavenging ability of SeNPs-CS/COS-Ms is the sum of the individual antioxidant capacities of their constituents, although the SeNP vehicle was considered as a whole. However, the natural activity of bare SeNPs to scavenge free radicals was difficult to determine due to its poor stability [10,11]. Here, the contribution of CS/COS to the free radical scavenging ability of SeNPs-CS/COS-Ms was also studied. As shown in Figure 4 and Table 3, the radical scavenging ability of CS/COS microspheres (CS/COS-Ms) was much weaker than that of SeNPs-CS/COS-Ms with the same CS:COS ratio. This result suggests that the contribution of CS or COS to the radical scavenging activity of SeNPs-CS/COS-Ms is limited. In other words, SeNPs might contribute greatly to the total radical scavenging ability of SeNPs-CS/COS-Ms.

### 3.5. Acute Toxicity of SeNPs-CS/COS-Ms

The safety of SeNPs-CS/COS-Ms should be considered before evaluating their potential in the clinic. As reported in previous work [10,11,45], SeNPs are safer than selenite or selenomethionine, which are responsible for the toxicity of SeNPs/vehicle complexes. In this study, SeNPs-CS/COS-Ms were found to be much safer than selenite in terms of Se dose, with a median lethal dose (LD_50_) of around 20-fold of that of sodium selenite in KM mice (Table 4). Considering the high safety of CS/COS-Ms (LD_50_ > 20 g/kg bw) and the low content of SeNPs (15.1 mg/g) within SeNPs-CS/COS-Ms, SeNPs might contribute greatly to the total toxicity of SeNPs-CS/COS-Ms. This result is consistent with our previous study [10] reporting that 18-fold of the LD_50_ (as compared with selenite) was achieved in Institute of Cancer Research (ICR) mice by embedding SeNPs into CS microspheres. Also, SeNPs loaded on bovine serum albumin (BSA) [45] or a chitosan/citrate complex [11] yielded a similar outcome. SeNPs loaded on a protein or polysaccharide may have comparable toxicity when (1) they share similar basic physicochemical properties [46,47], such as shape, size, chemical composition, and surface properties, and (2) they can escape from their vehicles in the stomach [11]. In addition to the recorded cumulative mortality, mice were dissected to investigate the damage to the body caused by the oral administrations. Hemorrhagia points in the lungs and an abnormal liver with a rough surface were found in the dead mice given selenite or SeNPs-CS/COS-Ms (Figure S2, Supplementary Materials), indicating that these two Se forms targeted the lungs and liver. However, normal organs in the surviving mice were found at the end of the 14-day acute toxicity test. This result suggests that the damage caused by SeNPs-CS/COS-Ms can be lessened over time. Taken together, these results suggest that SeNPs, when loaded onto CS/COS-based microspheres, could be used in the clinic due to their low toxicity and good release.

### 3.6. Protection of SeNPs-CS/COS-Ms against Oxidative Stress Induced by Alcohol

#### 3.6.1. Influence of SeNPs-CS/COS-Ms on Growth

Male KM mice were orally administered SeNPs-CS/COS-Ms at the doses of 40, 80, and 120 mg/kg bw (equal to 0.36%, 0.72%, and 1.09% of the LD_50_, respectively) before the 50% ethanol challenge test. The body weight was recorded to monitor the growth of the animals. As shown in Table 5 and Figure S3, little difference in growth was found among the mice throughout the experiment. The behavior and appearance of the mice in the other groups were similar to those in the normal control group, which was given normal saline. The tested compounds, including Vc, sodium selenite, CS/COS-Ms, and SeNPs-CS/COS-Ms, at their respective doses seemed to be safe to the mice during the experiment.

#### 3.6.2. Se Retention by SeNPs-CS/COS-Ms

**Figure 5:**
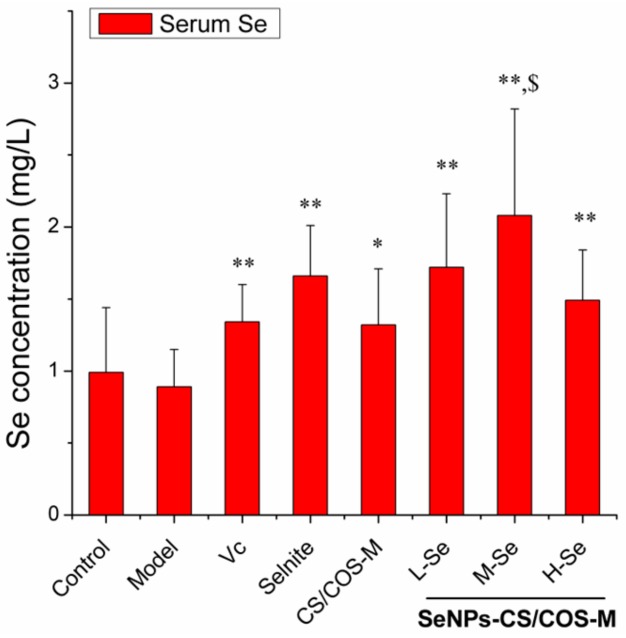
The serum Se retention within KM mice. The dose of each group is presented in Table 1. * *p* < 0.05, ** *p* < 0.01 versus the Model group; ^$^ *p* < 0.05 versus the CS/COS microsphere (CS/COS-M) group.

A low-Se diet (<0.1 mg Se/kg) may lead to Se deficiency in the body, and Se supplements can improve the Se level [10,11,48,49]. In this study, the serum Se retention in mice was found to be improved by selenite and SeNPs-CS/COS-Ms, while Vc and CS/COS-M might have also contributed to the Se deposition (Figure 5). It was evident that (1) SeNPs-CS/COS-Ms contributed to Se deposition more than CS/COS-M (*p* < 0.05), (2) Se retention occurred in a dose-dependent manner, and (3) the Se deposition of all of the SeNPs-CS/COS-Ms was comparable to that of selenite (*p* > 0.05) when their Se doses were equal (1.21 mg Se/kg bw). This implies that the SeNPs within the SeNPs-CS/COS-Ms made an important contribution to the Se retention. This result is consistent with that of our previous study [10] reporting that SeNPs, rather than their CS vehicle, contributed to the capacity of SeNPs-CS-Ms to supplement Se.

The SeNPs-CS/COS-Ms and selenite had comparable Se retention capacity, while selenite produced a higher level of serum Se in mice than the SeNPs-CS-Ms did [10]. It seems that the delivery of SeNPs by using the CS/COS system might benefit Se retention. This might partly be due to the improvement in the release of free SeNPs in the digestive tract when a high percentage of COS is introduced (Figure 3). Besides this, the dose of SeNPs needs more attention. The selenite and SeNPs were found to actually be the main Se supplement, in consideration of the extremely low Se content in the feed (<0.1 μg Se/g of diet) and the estimated daily intake of adult KM mice (about 4–8 g of diet/day each) [50]. These two Se sources might be at ‘super-nutritional’ levels, according to Barnes [48] and Raines [49]. This might be also be confirmed by the low statistical significance of the Se levels among the L-, M-, and H-Se groups (Figure 5). SeNPs-CS-Ms at the dose of 40 mg/kg bw (equally 0.6 mg Se/kg bw) were able to meet the requirement for adequate or super-nutritional Se. Thus, they deserve further investigation.

#### 3.6.3. Biomarkers

Excessive consumption of alcohol can lead to severe oxidative stress in animals and humans due to its toxicity and its ability to induce ROS in vivo [17,20,30,31]. Here, the antioxidant potential of SeNPs-CS/COS-Ms was evaluated in KM mice suffering from an alcohol challenge, and some biomarkers relevant to the antioxidant activity of SeNPs-CS/COS-Ms were investigated. As shown in Table 6 and Table 7, oral administration of alcohol resulted in strong oxidative stress characterized by an increase in TBARS and TCC, suggesting that oxidative damage occurred to the lipids and proteins in cells [51,52]. Alcohol was also found to decrease the levels of some protective factors, including GSH, GSH-Px, SOD, and CAT. However, SeNPs-CS/COS-Ms at the studied doses reduced TBARS and TCC, maintained GSH, and upregulated the levels of GSH-Px, SOD, and CAT, when compared with the model group given ethanol (*p* < 0.05 or *p* < 0.01). These results indicate that the SeNPs-CS/COS-Ms have a powerful antioxidant ability that is comparable to that of Vc (both 80 mg/kg bw).

Some points need attention. The first is the contributions of CS, COS, and SeNPs to the overall antioxidant capacity of SeNPs-CS/COS-Ms. CS/COS-Ms (80 mg/kg bw) did not enhance the abovementioned biomarkers, whereas SeNPs-CS/COS-Ms containing almost an equal amount of CS/COS were able to improve the antioxidant status in mice, as evidenced by the reduction in TBARS and TCC, the increase in GSH, and the upregulation of both SOD and GSH-Px (*p* < 0.05 or *p* < 0.01, versus Model). It was probably the SeNPs within SeNPs-CS/COS-Ms that contributed to the antioxidant activities of the SeNP supplement in vivo. The second point is the efficiency of SeNPs in boosting GSH-Px, which is a very important antioxidant enzyme family whose members use Se as an integral part of their catalytic sites [2,4]. As presented in Table 6, SeNPs-CS/COS-Ms at the doses of 80 and 120 mg/kg bw (equal to 1.2 and 1.8 mg Se/kg bw, respectively) prevented the ethanol-induced reduction of GSH-Px, whereas sodium selenite at the dose of 80 mg/kg bw (equal to 1.2 mg Se/kg bw) was unable to recover the level of GSH-Px. Considering the comparable Se retention among these Se sources (Figure 5), SeNPs in the form of SeNPs-CS/COS-M were more efficient in boosting the GSH-Px level than selenite. This result is not in line with some previous studies [10,53] reporting that comparable GSH-Px levels would be obtained if the Se doses were equal among different Se sources, such as selenite [10,53], BSA-SeNPs [53], and SeNPs-CS-Ms [10]. It is possible that the Se retention by SeNPs-CS/COS-Ms (shown in Figure 4) contributed to the Se storage pool in the body [54,55,56,57], finally leading to an enhancement in GSH-Px. Overall, SeNPs-CS/COS-Ms might protect animals from ethanol-induced oxidative risk, based on their capacity to upregulate the levels of GSH-Px, SOD, and CAT.

## 4. Conclusions

Both CS and COS can be used to synthesize spherical SeNPs; however, the former are superior to the latter in stabilizing SeNPs. The SeNPs-CS/COS-Ms were a collection of microparticles with a smooth or wrinkled surface and a hollow core, and SeNPs of around 50 nm were embedded in the SeNPs-CS/COS-Ms. Both the physical isolation of each SeNP during storage and the successful release of these nanoparticles in the stomach were carried out by SeNPs-CS/COS-Ms. The free radical scavenging ability of SeNPs-CS/COS-Ms was found to be acceptable and much stronger than that of selenite. This Se formulation was also found to be significantly more safe than selenite, with an LD_50_ of about 20-fold of that of selenite in mice. SeNPs-CS/COS-Ms might retard alcohol-induced oxidative stress by attenuating TBARS and TCC as well as by enhancing the levels of GSH, GSH-Px, SOD, and CAT. In summary, this mixed CS/COS microparticles design for SeNPs opens up a new path for oral delivery of Se with higher efficacy and better biosafety. SeNPs-CS/COS-Ms are a candidate source of Se worthy of further development for nutrient supplements or even nanomedicines that aim to defend against alcohol-induced oxidative injury.

## Figures and Tables

**Table 1:** Intragastric administration during the ethanol challenge experiment (Kunming (KM) mice, *n* = 10).

Group	Pre-Treatment (Once Daily, Day 1–35)	Treatment (Once, Day 36)
Control	Normal saline	Normal saline
Model	Normal saline	50% Ethanol (12 mL/kg bw)
Vc	Vc (80 mg/kg bw)	50% Ethanol (12 mL/kg bw)
Sodium selenite	Sodium selenite (2.65 mg/kg bw)	50% Ethanol (12 mL/kg bw)
CS/COS-M	CS/COS-M (80 mg/kg bw)	50% Ethanol (12 mL/kg bw)
L-Se	SeNPs-CS/COS-M (40 mg/kg bw)	50% Ethanol (12 mL/kg bw)
M-Se	SeNPs-CS/COS-M (80 mg/kg bw)	50% Ethanol (12 mL/kg bw)
H-Se	SeNPs-CS/COS-M (120 mg/kg bw)	50% Ethanol (12 mL/kg bw)

bw, body weight.

**Table 2:** The dynamic light scattering (DLS) results for bare SeNPs, COS-SeNPs, and CS-SeNPs (*n* = 3).

Sample	Z-Average Diameter (nm)	PDI
Bare SeNPs (fresh)	102.9 ± 6.4	0.355 ± 0.06
COS-SeNPs (fresh)	97.5 ± 3.6	0.305 ± 0.044
CS-SeNPs (fresh)	103.3 ± 2.3	0.262 ± 0.035
Bare SeNPs (after 6 h of storage at 25 °C)	265.2 ± 15.6	0.351 ± 0.040
COS-SeNPs (after 14 days of storage at 25 °C)	335.7 ± 11.7	0.472 ± 0.028
CS-SeNPs (after 14 days of storage at 25 °C)	80.5 ± 0.6	0.116 ± 0.018

PDI, polydispersity index.

**Table 3:** The EC_50_ of the free radical scavenging activities of the samples (mg/L, *n* = 3).

Free Radical	Vc	Sodium Selenite	CS/COS-M ^1^	SeNPs-CS/COS-M ^2^
DPPH	2.90 ± 0.04	>>103.8	>>600	29.54 ± 1.24
O_2_^−^	5.80 ± 1.00	>>138.4	1082 ± 104	34.45 ± 1.57

EC_50_, concentration for 50% of maximal effect. ^1^ The ratio of CS:COS was 10:90. ^2^ The selenium content was 15 mg/g, and the ratio of CS:COS was 10:90.

**Table 4:** Results of the acute lethal test by a single oral administration in KM mice (*n* = 10).

Sample	Se Content (mg/g)	LD_50_^#^ (mg/kg bw)	LD_50_ (Se) * (mg Se /kg bw)
Sodium selenite ^1^	456.7	19.2 (16.2–22.7) ^$^	8.8 (7.4–10.4) ^^^
CS/COS-M	--	>20 × 10^3^	--
SeNPs-CS/COS-M	15.1	11.06 × 10^3^ (6.28 × 10^3^ –19.51 × 10^3^) ^$^	167 (94.9–294.5) ^^^

^#^ LD_50_ = median lethal dose; * LD_50_ (Se) = LD_50_ × Se content. ^$^ The LD_50_ of the 95% confidence interval. ^^^ The LD_50_(Se) of the 95% confidence interval. ^1^ Reference [11].

**Table 5:** Body weight of KM mice (*n* = 10).

Group	Body Weight (g)		Increase (%)
	Day 1	Day 35	
Control	22.93 ± 1.45	43.56 ± 1.20	89.97
Model	23.08 ± 0.79	44.84 ± 3.63	94.28
Vc	23.38 ± 0.85	43.33 ± 3.98	85.33
Sodium selenite	22.85 ± 1.23	41.36 ± 4.18	81.01
CS/COS-M	22.55 ± 1.12	42.73 ± 3.94	89.49
L-Se	22.92 ± 0.66	44.10 ± 2.41	92.41
M-Se	23.12 ± 0.47	43.21 ± 3.85	86.89
H-Se	21.93 ± 1.05	41.21 ± 2.22	87.92

**Table 6:** Serum biomarkers of KM mice (*n* = 10).

Group	TBARS (nmol/mL)	SOD (U/mL)	GSH-PX (U/mL)	CAT (U/mL)
Control	10.7 ± 4.0	95.9 ± 25.5	576 ± 160	89.7 ± 8.2
Model	13.9 ± 6.7	82.9 ± 26.2	187 ± 186 ^##^	84.5 ± 8.0
Vc	13.1 ± 2.4	84.6 ± 22.1	431 ± 253 *	87.2 ± 8.3
Sodium selenite	12.8 ± 4.6	101 ± 14	343 ± 189	79.7 ± 16.7
CS/COS-M	11.9 ± 4.7	83.7 ± 25.2	375 ± 285	92.2 ± 10.0
L-Se	9.2 ± 3.2 *	108 ± 10 *	256 ± 171	84.9 ± 9.7
M-Se	9.2 ± 1.8 *	105 ± 12 *	451 ± 112 **	88.6 ± 9.3
H-Se	11.8 ± 2.5	57.3 ± 15.5 *	437 ± 196 **	72.9 ± 21.2 ^#^

^##^*p* < 0.01 versus the Control group; * *p* < 0.05, ** *p* < 0.01 versus the Model group.

**Table 7:** Hepatic biomarkers of KM mice (*n* = 10).

Group	TBARS (nmol/mg Prot)	GSH (mg/g Prot)	TCC (nmol/mg Prot)	SOD (U/mg Prot)	CAT (U/mg Prot)
Control	3.64 ± 2.03	2.91 ± 1.20	12.6 ± 5.8	90.8 ± 16.5	105 ± 36
Model	3.93 ± 2.55	2.01 ± 0.48 ^#^	20.1 ± 10.1 ^#^	85.0 ± 16.3	69.6 ± 47.5
Vc	2.71 ± 1.16	3.60 ± 1.12 **	14.5 ± 4.1	109 ± 16 **	123 ± 57 *
Sodium selenite	3.16 ± 1.29	3.77 ± 1.36 **	19.0 ± 6.6	107 ± 16 **	147 ± 43 **
CS/COS-M	3.56 ± 0.91	2.10 ± 0.34	22.7 ± 11.3	96.7 ± 17.0	90.9 ± 36.3
L-Se	3.04 ± 0.77	2.59 ± 0.33 **	13.4 ± 7.8	110 ± 18 **	128 ± 39 **
M-Se	2.74 ± 0.96	2.88 ± 0.90 *	7.3 ± 3.3 **	92.1 ± 8.0	100 ± 50
H-Se	1.70 ± 0.33 *	2.53 ± 0.47 *	8.9 ± 3.5 **	98.2 ± 11.1 *	100 ± 17

prot, protein. ^#^ *p* < 0.05 versus the Control group; * *p* < 0.05, ** *p* < 0.01 versus the Model group.

## Supplementary Materials

The following are available online at https://www.mdpi.com/1999-4923/12/1/43/s1, Figure S1: The SEM images of SeNPs when they were initially synthesized in (A) water, (B) aqueous COS (2.5 kDa), and (C) aqueous CS (37 kDa); Figure S2: Organ pathology induced by SeNPs-CS/COS-Ms or selenite in mice, with the small arrows indicating hemorrhagia points in lungs and the bigger arrow indicating the abnormal liver; Figure S3: The body weight of KM mice before ethanol challenge.

## Abbreviations

BSA	bovine serum albumin
bw	body weight
CAT	catalase
CS	chitosan
CS/COS-Ms	chitosan/chitooligosaccharide microparticles
COS	chitooligosaccharide
CS-SeNPs	chitosan-decorated selenium nanopartilcels
DLS	dynamic light scattering
DPPH	1,1-diphenyl-2-picrylhydrazyl
EC_50_	concentration for 50% of maximal effect
EDS	energy-dispersive X-ray spectroscopy
GSH	glutathione
GSH-Px	glutathione peroxidase
ICP-MS	inductively coupled plasma mass spectrometry
ICR	Institute of Cancer Research
KM	Kunming
KMnO_4_	potassium permanganate
LD_50_	median lethal dose
MW	molecular weight
MWCO	molecular weight cut-off
PDI	polydispersibility index
RH	relative humidity
ROS	radical oxygen species
Se	selenium
SEM	scanning electron microscopy
SeNPs	selenium nanoparticles
SeNPs-CS/COS-Ms	selenium-nanoparticles-loaded chitosan/chitooligosaccharide microparticles
SeNPs-CS-Ms	selenium nanoparticles-embedded chitosan microspheres
SGF	simulated gastric fluid
SIF	simulated intestinal fluid
SOD	superoxide dismutase
SPF	specific-pathogen-free
TBARS	thiobarbituric acid-reactive substances
TCC	total carbonyl compounds
TEM	transmission electron microscopy
UF	ultrafiltration
Vc	ascorbic acid
