# Supplementary material for: Selenium-Nanoparticles-Loaded Chitosan/Chitooligosaccharide Microparticles and Their Antioxidant Potential: A Chemical and In Vivo Investigation
Source: Pharmaceutics. 2020 Jan 3;12(1):43. doi: 10.3390/pharmaceutics12010043 (PMC7022253; doi:10.3390/pharmaceutics12010043)
Supplement: Supplementary file 1 [file pharmaceutics-12-00043-s001.pdf]

pharmaceutics

Supplementary Materials: Selenium-Nanoparticles-Loaded Chitosan/Chitooligosaccharide Microparticles and Their Antioxidant Potential: A Chemical and in Vivo Investigation

Kaikai Bai*, Bihong Hong, Wenwen Huang and Jianlin He

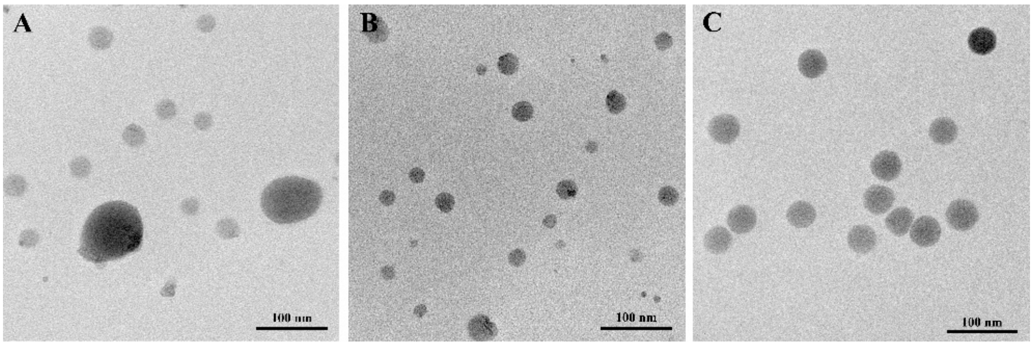

Figure S1. The SEM images of SeNPs when they were initially synthesized in (**A**) water, (**B**) aqueous COS (2.5 kDa) and (**C**) aqueous CS (37 kDa).

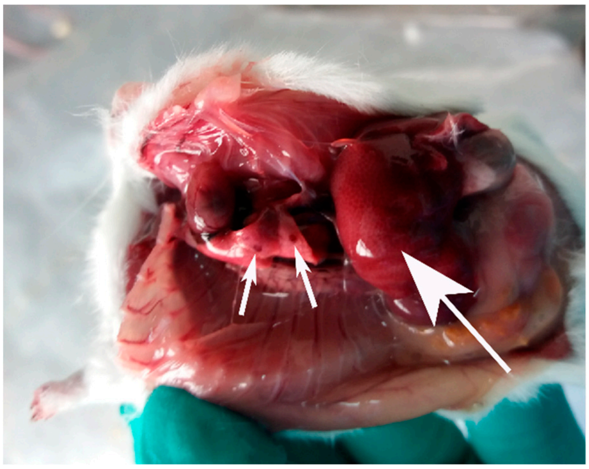

Figure S2. Organ pathology induced by SeNPs-CS/COS-Ms or selenite in mice, with the small arrows indicating hemorrhagia points in lungs and the bigger arrow indicating the abnormal liver.

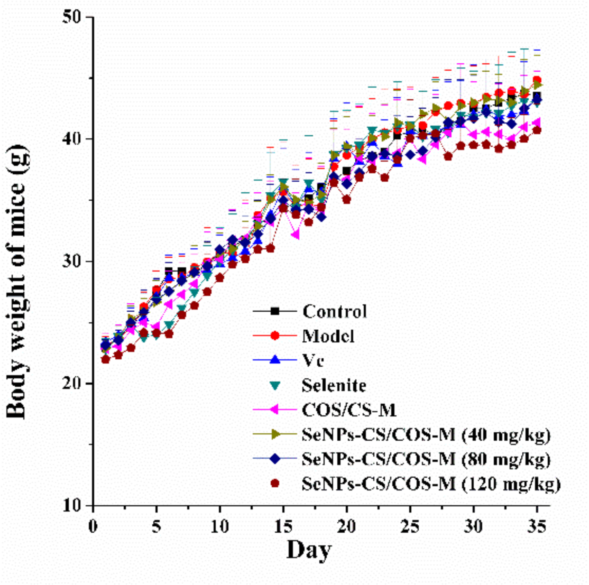

Figure S3. The body weight of KM mice before ethanol challenge.
